# Ultrafast Thermalization Pathways of Excited Bulk and Surface States in the Ferroelectric Rashba Semiconductor GeTe

**DOI:** 10.1002/adma.202200323

**Published:** 2022-05-12

**Authors:** Oliver J. Clark, Indrajit Wadgaonkar, Friedrich Freyse, Gunther Springholz, Marco Battiato, Jaime Sánchez‐Barriga

**Affiliations:** ^1^ Helmholtz‐Zentrum Berlin für Materialien und Energie Elektronenspeicherring BESSY II, Albert‐Einstein‐Str. 15 12489 Berlin Germany; ^2^ Nanyang Technological University Nanyang Link 21 Singapore 637371 Singapore; ^3^ Institut für Physik und Astronomie Universität Potsdam Karl‐Liebknecht‐Str. 24/25 14476 Potsdam Germany; ^4^ Institut für Halbleiter‐ und Festkörperphysik Johannes Kepler Universität A‐4040 Linz Austria; ^5^ IMDEA Nanoscience C/ Faraday 9, Campus de Cantoblanco Madrid 28049 Spain

**Keywords:** ferroelectric semiconductors, Rashba effect, spin‐ and angle‐resolved photoemission, spin–orbit coupling, time‐resolved photoemission, ultrafast dynamics

## Abstract

A large Rashba effect is essential for future applications in spintronics. Particularly attractive is understanding and controlling nonequilibrium properties of ferroelectric Rashba semiconductors. Here, time‐ and angle‐resolved photoemission is utilized to access the ultrafast dynamics of bulk and surface transient Rashba states after femtosecond optical excitation of GeTe. A complex thermalization pathway is observed, wherein three different timescales can be clearly distinguished: intraband thermalization, interband equilibration, and electronic cooling. These dynamics exhibit an unconventional temperature dependence: while the cooling phase speeds up with increasing sample temperature, the opposite happens for interband thermalization. It is demonstrated how, due to the Rashba effect, an interdependence of these timescales on the relative strength of both electron–electron and electron–phonon interactions is responsible for the counterintuitive temperature dependence, with spin‐selection constrained interband electron–electron scatterings found both to dominate dynamics away from the Fermi level, and to weaken with increasing temperature. These findings are supported by theoretical calculations within the Boltzmann approach explicitly showing the opposite behavior of all relevant electron–electron and electron–phonon scattering channels with temperature, thus confirming the microscopic mechanism of the experimental findings. The present results are important for future applications of ferroelectric Rashba semiconductors and their excitations in ultrafast spintronics.

## Graphical abstract

A time‐, spin‐, and angle‐resolved photoemission study of the ferroelectric Rashba semiconductor α‐GeTe is performed following ultrafast optical excitation. The thermalization pathways exhibit an unconventional temperature dependence, which is understood in terms of the underpinning microscopic scattering channels. The Rashba effect is found to inhibit electron–electron scatterings, enabling control of the overall dynamics with lattice temperature on subpicosecond timescales.

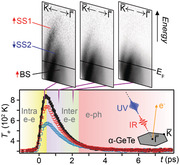

## 1 Introduction

Understanding the elementary scattering processes which underlie the relaxation of spin‐polarized carriers in narrow‐gap semiconductors with strong spin–orbit coupling is essential for future applications in spintronics.^[^ ^1^, ^2^, ^3^, ^4^, ^5^, ^6^, ^7^, ^8^ ^]^ A central challenge is to exploit the spin–orbit interaction to achieve efficient processing and storage of information without external magnetic fields.^[^ ^6^, ^7^, ^8^, ^9^, ^10^, ^11^, ^12^ ^]^ The spin–orbit interaction can cause a large Rashba effect when an inversion asymmetry occurs at the surface or interfaces, or if it is present in the bulk.^[^ ^13^, ^14^, ^15^, ^16^, ^17^ ^]^ As a result, the spin degeneracy of electronic states is lifted and their spin splitting becomes Δ*E* = 2α_R_|*k*|, which to first order depends linearly on momentum |*k*| and on the strength of the Rashba effect, as represented by the so‐called Rashba parameter α_R_.^[^ ^18^, ^19^ ^]^ A large Rashba effect is considered to be the key to achieving enhanced control of spin‐polarized currents,^[^ ^20^, ^21^ ^]^ efficient spin injection^[^ ^10^, ^22^ ^]^ and spin‐to‐charge interconversion,^[^ ^23^, ^24^, ^25^, ^26^ ^]^ large spin–orbit torques,^[^ ^5^, ^27^ ^]^ and slow carrier recombination,^[^ ^28^ ^]^ as well as to realizing topological superconductivity and Majorana fermions,^[^ ^6^, ^29^ ^]^ each distinct efforts in the multipronged approach toward the development of functional spintronic devices.

Of particular interest is the understanding and exploration of nonequilibrium properties of Rashba systems and their nanostructures.^[^ ^6^ ^]^ In this context, optical excitation by femtosecond (fs)‐laser pulses is an indispensable tool which could be utilized for more efficient and faster processing of spin information in future optical devices.^[^ ^30^ ^]^ A large Rashba effect is required for device miniaturization and to suppress spin randomizing scattering events known to occur in conventional semiconductors with small, or zero, Rashba splittings.^[^ ^31^ ^]^ Therefore, special attention is being devoted to understanding the influence of the Rashba spin texture on the relevant scattering channels which determine the recombination time scales of photoexcited carriers in systems with a large Rashba parameter.^[^ ^32^, ^33^, ^34^, ^35^ ^]^ In particular, identifying the thermalization and scattering pathways of ultrafast information transfer in these materials is critically important to overcome the performance limits of currently available devices. For instance, the excitation of spin–orbit systems with fs‐laser pulses has provided access to the ultrashort time scales involved in the elementary scattering processes underlying nonequilibrium giant spin injection,^[^ ^36^, ^37^ ^]^ light‐induced spin current generation,^[^ ^38^ ^]^ or all‐optical spin‐to‐charge conversion^[^ ^39^ ^]^ via the inverse Edelstein effect on the surface or the inverse spin Hall effect in the bulk.^[^ ^40^, ^41^ ^]^ Equally important, long‐lived spin polarization of optically excited carriers is an essential requirement to achieve efficient transfer of spin information over macroscopic distances in future devices.

Since the observation of a Rashba effect by angle‐resolved photoemission spectroscopy (ARPES) from metal surfaces,^[^ ^42^ ^]^ the tremendous progress in material synthesis has led to the discovery of a vast number of spin–orbit systems with increasingly large Rashba splittings not only at their surfaces or interfaces, but also in the bulk.^[^ ^17^ ^]^ In this respect, spin‐resolved ARPES has played a central role in the confirmation of both the Rashba splitting and associated chiral, momentum‐locked, spin texture.^[^ ^17^ ^]^ A prominent example of materials with a giant Rashba splitting are the polar semiconductors BeTeX (X = I, Cl, and Br).^[^ ^13^, ^43^ ^]^ Most recently, a different material class named ferroelectric Rashba semiconductors,^[^ ^12^ ^]^ such as rhombohedral germanium telluride (α‐GeTe), has come into focus.^[^ ^26^ ^]^ α‐GeTe is one of the few known binary ferroelectric semiconductors with a narrow bandgap, and possesses one of the largest observed Rashba parameters to date.^[^ ^12^ ^]^ As such, the unique combination of ferroelectricity and Rashba‐type spin–orbit effects in this material is considered an ideal platform for developing multifunctional devices with advanced memory and computing capabilities.

Considerable work has been carried out to characterize the electronic band structure of α‐GeTe in equilibrium. A giant Rashba splitting due to spin–orbit coupling and inversion symmetry breaking both at the surface and in the bulk has been demonstrated in previous theoretical and experimental studies.^[^ ^12^, ^14^, ^44^, ^45^, ^46^, ^47^, ^48^ ^]^ Spin‐resolved ARPES measurements have confirmed the predicted Rashba splitting of surface and bulk states,^[^ ^44^, ^45^, ^46^, ^47^ ^]^ as well as the reversibility of the spin texture with the ferroelectric polarization.^[^ ^49^, ^50^ ^]^ It was also shown how these unique properties of the α‐GeTe band structure can be used to achieve large spin‐to‐charge conversion^[^ ^26^, ^51^ ^]^ and its ferroelectric switching.^[^ ^26^ ^]^ However, very little is known about its nonequilibrium properties on ultrashort time scales. A direct observation of the nonequilibrium band structure of α‐GeTe following fs‐laser excitation, which is highly relevant not only to fundamentally understand the dynamics of excited bulk and surface states but also for the implementation of advanced functionalities in future ultrafast optical devices, remains elusive.

Here, we investigate the mechanisms of ultrafast energy transfer in the nonequilibrium band structure of α‐GeTe following optical excitation. To this end, we perform time‐resolved ARPES experiments to directly visualize the ultrafast response of the bulk and surface Rashba bands to optical excitation with fs‐laser pulses. Our data reveal a complex interplay between bulk and surface dynamics which due to the large Rashba splitting of the bands causes a bottleneck in the interband thermalization and relaxation processes of excited carriers. We further identify the dominant role of spin‐dependent electron–electron scattering processes by the observation of a counter‐intuitive temperature dependence of the thermalization pathways. The experimental results are supported by theoretical calculations within the Boltzmann approach including all types of elementary scatterings. The present findings are important for potential applications of ferroelectric Rashba semiconductors in ultrafast spintronics.

## 2 Results and Discussion

### 2.1 Electronic Properties of Transiently Occupied Bulk and Surface States

**Figure 1:**
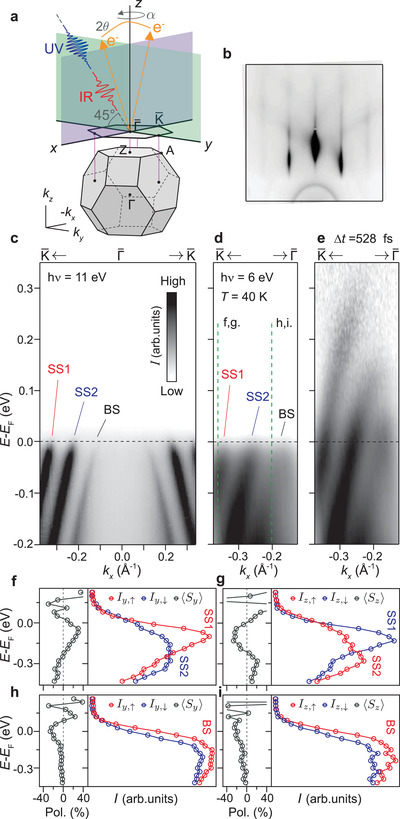
Sample characterization and electronic properties of excited states. a) Experimental geometry. b) Reflection high‐energy electron diffraction image of clean α‐GeTe. c,d) Energy–momentum dispersions of bulk (BS) and surface states (SS1, SS2) measured in equilibrium using synchrotron light of *hν* =11 eV photon energy (c) and fs‐laser 6 eV photons (d). e) Corresponding energy–momentum dispersion to the one in (d) at a time delay of Δ*t* = 528 fs after optical excitation by the pump pulse. f–i) Spin‐resolved energy distribution curves (red/blue circles for spin up/down, respectively) and spin polarizations (gray circles) corresponding to the chiral (S_*y*_) and out‐of‐plane (S_*z*_) spin components of surface (f,g) and bulk (h,i) states, taken at the momentum positions indicated in (d).

To access the nonequilibrium band dispersion of excited states in α‐GeTe and its temporal evolution beyond the Fermi level (*E*_F_), we performed time‐resolved ARPES (tr‐ARPES) measurements using infrared pump (1.5 eV) and ultraviolet probe (6 eV) fs‐laser pulses under the experimental geometry shown in **Figure** **1**a. The experiments were performed on 0.5 μm‐thick α‐GeTe(111) films grown by molecular beam epitaxy on BaF_2_(111) substrates (see Experimental Section for details). The high quality of the pristine α‐GeTe surface was confirmed by the presence of Kikuchi lines in reflection high‐energy electron diffraction, as seen in Figure 1b.

Figure 1c,d shows the energy–momentum band dispersion of α‐GeTe (111) measured by ARPES in equilibrium (i.e., no pump photons and probe photon energies of 11 and 6 eV, respectively) at a temperature *T* = 40 K along the $\bar{\Gamma }$–$\bar{K}$ direction of the surface Brillouin zone, depicted in Figure 1a alongside that of the bulk. The spectrum in Figure 1c was acquired using synchrotron light to access a larger energy–momentum region of the band dispersion while keeping similar experimental conditions as for the laser‐based ARPES measurements of Figure 1d. One can identify distinct and well‐separated features up to *E*_F_ corresponding to spin‐polarized surface and bulk‐like states exhibiting hole‐like behavior (denoted as SS1, SS2, and BS, respectively). The characteristic dispersion of the Rashba‐split sub‐bands SS1 and SS2 forming the surface state can be clearly seen crossing *E*_F_, where we derive a giant momentum splitting of Δ*k*_∥_ = 0.11 Å^−1^ as expected for a Te‐terminated surface with an outward ferroelectric polarization.^[^ ^14^ ^]^ Under these conditions, we do not observe intensity contributions from surface‐resonance states dispersing away from the $\bar{\Gamma }$ point and overlapping with bulk valence‐band states at off‐normal wave vectors.^[^ ^44^ ^]^ Together with the enhanced bulk sensitivity at low probe photon energies,^[^ ^52^ ^]^ this suggests that bulk‐like BS states in Figure 1 possess a strong and distinct bulk character. This assignment appears consistent with previous calculations of the electronic band structure of α‐GeTe in equilibrium, not only concerning the dispersion and energy positions of the bands, but also their degree of surface localization.^[^ ^14^ ^]^

The surface character of the SS1 and SS2 bands can be further recognized in Figure 1c,d by the fact that their overall dispersion and thus their momentum splitting at *E*_F_ remain unchanged when reducing the photon energy from 11 to 6 eV. In contrast, owing to the dispersive nature of bulk states with photon energy or wave vector perpendicular to the surface (*k*_*z*_),^[^ ^52^ ^]^ the momentum separation at *E*_F_ between the bulk‐like BS and the inner SS2 band decreases from 0.09 Å^−1^ at 11 eV (Figure 1c) to 0.07 Å^−1^ at 6 eV (Figure 1d). Note that due to the *k*_*z*_‐dispersion of BS states, this separation becomes progressively smaller away from *E*_F_ in Figure 1d, and in consequence, their corresponding group velocity changes from ≈2.74 eVÅ in Figure 1c to ≈2.29 eVÅ in Figure 1d.

Taking into account that the lattice constant of α‐GeTe along the *z* direction is *c* = 5.98 Å and using the empirical inner potential *U*_0_ = 8.5 eV,^[^ ^47^ ^]^ we derive that at a 6 eV photon energy we probe a *k*_*z*_ value of ≈1.62 Å^−1^, which is in the immediate vicinity of the *Z* point of the bulk Brillouin zone projecting onto $\bar{\Gamma }$ (see Figure 1a). Therefore, with 6 eV probe fs‐laser pulses here we follow the energy–momentum dispersion of bulk states very close to the *Z*‐*A* direction along which the bulk valence band reaches its maximum,^[^ ^12^, ^14^, ^44^, ^45^, ^46^, ^47^ ^]^ which in our case is located above *E*_F_ due to the *p*‐type doping caused by Ge vacancies.^[^ ^53^ ^]^ This can be seen in Figure 1e, where the unoccupied part of the band dispersion above *E*_F_ in Figure 1d is now transiently populated with excited electrons at a delay time Δ*t* = 528 fs after optical excitation by the pump pulse. In Figure 1e, one can also distinguish the dispersion of the surface Rashba bands up to energies of ≈0.3 eV above *E*_F_, indicating that the dynamics of excited electrons at higher energies proceeds on a shorter time scale. By following the band dispersion of SS1 and SS2 states above *E*_F_, we systematically derive an energy splitting Δ*E* = 281 meV that is consistent with a Rashba parameter α_R_ = 2*E*_R_/*k*_R_ = 5.1 eVÅ, where twice the Rashba energy is defined as 2*E*_R_ = Δ*E* and *k*_R_ = Δ*k*_∥_/2 = 0.055 Å^−1^ is the Rashba momentum. This value of α_R_, to the best of our knowledge, is the largest observed in this material to date, exceeding the one found in previous photoemission experiments so far accessing only occupied states.

To understand if the interaction between surface and bulk‐like states influences their spin texture, we performed spin‐resolved ARPES measurements at selected momentum cuts across the dispersion of different states, as indicated by dashed vertical lines in Figure 1d. In Figure 1f–i, we show the corresponding spin‐resolved energy distribution curves (red/blue circles for spin up/down, respectively) and spin polarizations (gray circles) measured at 6 eV for both surface (Figure 1f,g) and bulk (Figure 1h,i) states. Besides the large spin splitting, the alternating orientation of the in‐plane chiral spin components of SS1, SS2 (Figure 1f), and BS states (Figure 1h) is consistent with that predicted for a Te‐terminated surface,^[^ ^14^ ^]^ which features a counterclockwise (clockwise) chiral spin texture for SS2 (SS1 and BS) states. The in‐plane spin polarization of the bulk‐like BS and the inner SS2 surface band is considerably smaller than that of outer SS1 surface band. This is accompanied by an out‐of‐plane canting of the electron spins in the direction perpendicular to the surface (Figure 1g,i), as theoretically predicted.^[^ ^14^ ^]^ The reduced spin polarization in conjunction with the collinear out‐of‐plane spin canting for SS2 and BS states indicates an influence of interband spin hybridization on the spin polarization.^[^ ^54^ ^]^ This raises questions about the exact role of spin‐dependent interband scattering processes in the relaxation dynamics of excited states, which has been predicted to be exceptionally slow due to the large Rashba splitting of the bands.^[^ ^28^, ^55^ ^]^

### 2.2 Ultrafast Response of Surface and Bulk States to Laser Excitation

**Figure 2:**
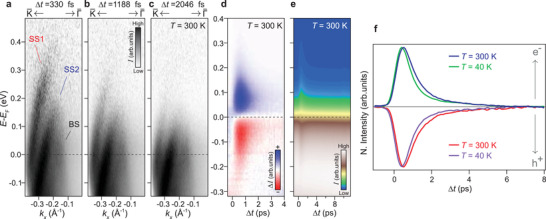
Ultrafast dynamics of excited bulk and surface states. a–c) Energy–momentum dispersions of α‐GeTe acquired by time‐ and angle‐resolved photoemission at a temperature *T* = 300 K at selected time delays following optical excitation. Surface‐state subbands and bulk‐like states are denoted as SS1, SS2, and BS, respectively. d) Momentum‐integrated difference spectra after subtracting the equilibrium band dispersion at a time delay Δ*t* = −1 ps before optical excitation. The blue and red color scale represents the excited population of electrons and holes, respectively. The position of the Fermi level (*E*_F_) is highlighted by horizontal dashed lines. e) Momentum‐integrated spectrum corresponding to the one shown in (d) displayed as total intensity in a wider range of pump–probe time delays. f) Normalized tr‐ARPES intensities integrated over the energy window above (below) *E*_F_ in (e) at different temperatures for excited electrons (holes). Blue and red (green and violet) solid lines correspond to measurements of the transient populations of electrons and holes at *T* = 300 K (40 K), respectively.

**Figure 3:**
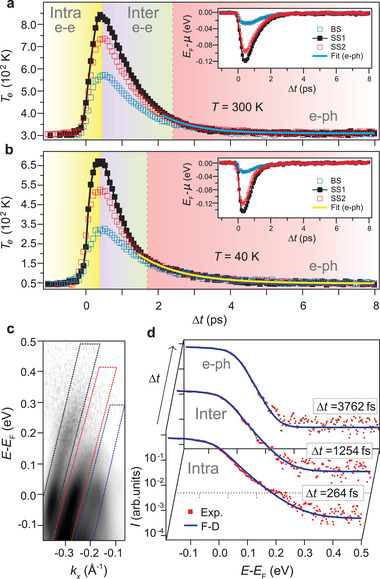
Thermalization dynamics of photoexcited states in α‐GeTe. a,b) Temporal evolution of the transient electronic temperatures for bulk (BS) and surface states (SS1 and SS2) at a sample temperature of *T* = 300 K (a) and 40 K (b). The corresponding transient chemical potentials are given in the insets. Blue (black and red) traces correspond to BS (SS1 and SS2) states. Three distinct regions predominantly associated with intraband and interband thermalization via electron–electron scatterings and energy dissipation into the lattice via electron–phonon scatterings are highlighted. The blue solid line in (a) (yellow in (b)) is an exponential fit to a single electronic temperature in the region dominated by electron–phonon scatterings. c) Nonequilibrium band dispersion highlighting the energy–momentum windows in which the electronic distribution associated to SS1 (black), SS2 (red) and BS (blue) states is fit to a Fermi–Dirac distribution at each time delay. d) Representative energy‐distribution curves (red squares) for SS1 states at selected time delays in the different thermalization stages highlighted in (a), and corresponding fits to Fermi–Dirac distributions (blue solid lines).

To identify the most relevant elementary scattering channels determining the relaxation and thermalization time scales of excited surface and bulk states, we investigated their temporal evolution both at room and low temperature. **Figure** **2**a–c shows several tr‐ARPES energy–momentum dispersions acquired at *T* = 300 K at selected time delays following optical excitation by the pump pulse. Similar to the tr‐ARPES dispersion in Figure 1e at *T* = 40 K, these spectra are representative snapshots of the nonequilibrium band dispersion sliced through a 3D data volume from which the results in Figures 2d–f and 3 are derived.

In Figure 2a, the initial population of excited SS1 states at higher energies can be clearly observed in an energy window of about 0.2–0.4 eV above *E*_F_. The overall dispersion of bulk and surface states is consistent with the one in Figure 1e, meaning that the temperature does not influence their relative energy positions with respect to *E*_F_ or the large Rashba splitting of the bands. The population of higher‐energy SS1 states slowly relaxes down to energies slightly above the top of the SS2 dispersion on a time scale of more than 1 ps (Figure 2b). Despite the reduced intensity at smaller wave vectors, the bending back of the hole‐like dispersion of SS2 states above *E*_F_ can also be distinguished in Figure 1e, where, as expected, the momentum splitting between SS1 and SS2 states increases slightly as the top of the SS2 band is approached. After ≈2 ps, the SS1 and SS2 populations have energetically relaxed toward an energy close to the BS band maximum, and excited electrons within surface and bulk states decay according to similar dynamics (Figure 2c). This behavior indicates that there is a continuous electron transfer between the different bands, and that their dynamics at lower energies is affected by interband scattering processes from higher‐lying states.

The tr‐ARPES spectra in Figure 2a–c resemble the temporal evolution of a continuously thermalizing distribution of hot electrons for SS1, SS2, and BS states. The whole electronic system slowly returns back to equilibrium on a longer time scale of several picoseconds, as can be seen in the momentum‐integrated tr‐ARPES spectra of Figure 2d,e. In the corresponding difference spectra displayed as Δ*I*(*E*,*t*) = *I*(*E*,*t*) − *I*(*E*,−1 ps) in Figure 2d, one can observe a narrow transient distribution of electron and holes within ≈300 meV above and below *E*_F_, respectively. Within our experimental time resolution of δ*t* = 160 fs, the bulk Rashba spin subbands dispersing further below *E*_F_ remain occupied and do not contribute to the observed transient distribution.

The interplay of electron–electron and electron–phonon elementary scattering events can drive both carrier relaxation and thermalization.^[^ ^56^, ^57^, ^58^, ^59^, ^60^ ^]^ The lack of higher energy excitations, which can be also recognized at the onset of the optical excitation in Figure 2e, indicates that an increasing contribution of faster electron–electron scattering processes as the energy increases is one of the most important mechanisms during the initial stages of thermalization. This is also supported by the fact that twice the maximum (minimum) energy of the electron (hole) distribution at early delay times is about the size of the bandgap, which in α‐GeTe is around 0.61–0.63 eV.^[^ ^14^ ^]^ At longer time delays, the energy stored by the excited electrons is gradually released into the lattice until both systems eventually equilibrate within about 8 ps (Figure 2f). The total carrier recombination time both at *T* = 300 K and 40 K is more than an order of magnitude longer than in metals (<0.3 ps), suggesting that the main elementary scattering channels which determine the dynamics of bulk and surface states are constrained by spin selection due to the complex alternating spin texture of the bands (Figure 1f–i). While electron–electron scatterings can cause carrier relaxation by redistributing energy between higher‐ and lower‐energy states, only electron–phonon scatterings reduce the total energy stored by the excited electrons. However, electron–electron scatterings can also influence the rate at which the energy is released into the lattice because, due to energy conservation, they produce secondary electron excitations which increase the number of electrons emitting phonons.^[^ ^58^ ^]^

From the temporal evolution of the tr‐ARPES intensities integrated over the entire energy window above (below) *E*_F_ for excited electrons (holes) in Figure 2f, we derive that the transient carrier distribution relaxes toward equilibrium with an energy‐averaged time constant of τ_E_ = 1.2 ps at *T* = 300 K. The relaxation at *T* = 40 K proceeds at a higher rate according to a smaller time constant of τ_E_ = 0.8 ps. Note that the value of τ_E_, which corresponds to the time required for the total distribution to drop by a factor of 1/*e*, represents a global time scale that, to a first approximation, provides information about the characteristic relaxation rate of the whole electronic system and not necessarily the time scale associated with an individual relaxation channel.

The slower relaxation at higher temperature is counter‐intuitive and difficult to reconcile with the faster dynamics that is instead expected due to the higher electron–phonon scattering rate at elevated lattice temperature, both at the surface and in the bulk.^[^ ^55^, ^61^ ^]^ Earlier on, the relaxation dynamics in α‐GeTe has been theoretically predicted to become slower with decreasing temperature due to the combined effect of the Rashba spin texture and a lower electron–phonon scattering rate as temperature decreases.^[^ ^55^ ^]^ The different behavior in Figures 2 and 3 indicates that the ultrafast dynamics in α‐GeTe, as observed here, is possibly the consequence of a more complex interplay between spin texture and several coexisting electron–electron and electron–phonon relaxation channels between the surface and the bulk. The fact that the overall dynamics is faster than theoretically predicted pinpoints the importance of bulk‐to‐surface scattering processes, which so far have not been considered in previous theoretical models, in both the relaxation and thermalization of excited states.

### 2.3 Thermalization Dynamics of Spin‐Polarized Bulk and Surface Carriers

To further understand if spin‐dependent scattering processes between surface and bulk Rashba states play an important role in the dynamics, we examined the ultrafast temporal evolution of the transiently excited carrier populations within different bands. To this end, we analyzed separately the energetic distribution of spectral weight for the surface and the bulk. This is shown in **Figure** **3**, where the transient electronic temperatures (Figure 3a,b) and chemical potentials (insets) of the hot electronic populations of SS1, SS2, and BS states at different sample temperatures are obtained by fitting the energy‐distribution curves extracted in their respective energy–momentum regions (Figure 3c) to a Fermi–Dirac distribution at each time delay (Figure 3d).

We note that for the pump intensities employed here (≈6.3 × 10^8^ W cm^−2^), the energy density spread across a thermal carrier distribution within few tens of nanometers from the surface is much smaller than the energy of photoexcited carriers.^[^ ^58^ ^]^ Thus, here we discuss the intraband and interband thermalization processes of transient Rashba states in a fixed electronic structure where the electron–electron and electron–phonon coupling strengths are not affected by the pump excitation itself.^[^ ^58^ ^]^ This is critically important to avoid a coupling to coherent‐phonon oscillations,^[^ ^62^ ^]^ which might lead to extremely small, almost undetectable changes of the Rashba splitting, and in consequence impede to exploit the strength of the fundamental electron–electron interactions in the ground‐state to achieve a large tunability of the carrier lifetimes following ultrafast optical excitation.

Turning attention to the temporal evolution of the transient electronic temperatures of the hot bulk and surface populations in Figure 3a, one can see that, while after optical excitation SS1, SS2, and BS states quickly reach intraband thermalization, the equilibration of their electronic temperatures occurs on a longer time scale. In particular, the electronic temperatures begin at a lattice temperature of 300 K at Δ*t* = −1 ps, reach a different maximum value at ≈350 fs when intraband thermalization is established, and equilibrate at longer time delays within about 2.4 ps. In this configuration, the corresponding transient chemical potentials have been equilibrated (inset in Figure 3a), and the electronic system has undergone interband thermalization. Following this process, the SS1, SS2, and BS populations progressively cool down with the same temperatures and chemical potentials until the electronic system returns back to equilibrium by transferring energy to the lattice.

At *T* = 40 K, as one can see in Figure 3b, the thermalization dynamics proceeds on different time scales than at room temperature. While the maximum relative increase of the electronic temperature within each band is similar to Figure 3a, the temperature rise is faster, and intraband thermalization is achieved on a slightly shorter time scale within about 260 fs. Moreover, the electronic temperatures and chemical potentials are equilibrated at earlier time delays, and interband thermalization is established significantly faster, in about 1.5 ps. In contrast, after the temperatures and chemical potentials have been equilibrated, the subsequent cooling of the electronic populations proceeds at a slightly lower rate than at room temperature. Consistent with an electronic cooling predominantly associated with electron–phonon scatterings that become more effective at elevated lattice temperature,^[^ ^61^ ^]^ an exponential fit to a single electronic temperature in this region of longer time delays yields the time constants τ_ep_ = 0.93(4) ps at 300 K (Figure 3a) and τ_ep_ = 1.17(5) ps at 40 K (Figure 3b).

Strikingly, the opposite temperature dependence is observed for both intraband and interband thermalization. Electron–electron scatterings are commonly thought to be a very efficient mechanism that causes ultrafast thermalization not only within a single band, but also between bands, on a typical time scale of few tens of fs.^[^ ^63^ ^]^ It should be noted, however, that in the most general case, both electron–electron and electron–phonon scatterings can drive intraband and interband thermalization.^[^ ^58^ ^]^ Despite that at 300 K, in Figure 3a, intraband thermalization proceeds on a longer time scale than the laser excitation, the shorter electron–phonon scattering time at higher lattice temperature, in conjunction with the small energy of the phonons (≈13 meV^[^ ^64^ ^]^), is not compatible with a faster intraband thermalization at lower lattice temperature. Furthermore, while electron–phonon scatterings have less strict selection rules than electron–electron scatterings, both are constrained by spin selection,^[^ ^60^ ^]^ with spin‐flip scatterings being the less probable process as long as time‐reversal symmetry is maintained.^[^ ^55^ ^]^ Therefore, it is clear that large‐*k*‐transfer intraband transitions via electron–phonon scatterings have extremely low efficiency due to the chiral spin texture of excited states, and that low‐*k*‐transfer electron–phonon scatterings are not the mechanism responsible for intraband thermalization in the present case.

Similarly, the reduced efficiency with which electron–phonon scatterings can establish thermal equilibrium between SS1, SS2, and BS states is not compatible with the counter‐intuitive interband thermalization process that we observe. The reason is potentially threefold. First, due to the fact that the maximum phonon energy that can be exchanged in each interband transition is very small, electron–phonon scatterings do not have a direct thermalization channel in which to redistribute energy between highly excited SS1 or SS2 states far above *E*_F_ and lower‐lying BS states. Second, low‐*k*‐transfer electron–phonon interband scatterings are significantly suppressed due to the alternating spin texture of the bands and because 2*E*_R_ ≫ 13 meV. Third, interband electron–phonon scatterings which require large‐*k* transfers to overcome twice the Rashba momentum are Pauli blocked at 40 K because *k*_B_ *T* = 3.4 meV$<\hbar \omega_{2k_{R}}$ = 2*ℏc*_p_ *k*_R_ = 5.7 meV, where *k*_B_ is the Boltzmann constant and *c*_p_ = 7.9 × 10^3^ m s^−1^ is the typical speed of sound associated with a linear acoustic phonon dispersion.^[^ ^64^ ^]^ Therefore, our results indicate that we observe an unusual temperature dependence which is inherent to elementary electron–electron scattering processes of extremely low efficiency, and that the thermalization dynamics in α‐GeTe is completely different than what conventional wisdom stipulates, which is that in the majority of the cases electron–electron scatterings are only relevant during the ultrashort laser excitation.

### 2.4 Influence of Spin‐Dependent Electron–Electron Scatterings on the Thermalization and Relaxation Pathways

The fundamental interactions responsible for the thermalization of the whole electronic system require the excited electronic populations of bulk and surface states to be able to exchange energy through scattering. In this respect, the effectiveness of the elementary scattering processes that lead to the global thermalization and relaxation of the electronic populations depends not only on the dispersion and spin texture of excited states, but also on the scattering probability, which is related to the strength of the underlying interaction. All these properties are key ingredients determining the relevant time scales of charge and energy transfer. In simple band systems where electron–electron scatterings are very strong, global thermalization via electron–electron scatterings can be achieved extremely fast, and it is usually considered, in a simple approximation, instantaneous.^[^ ^58^, ^63^, ^65^ ^]^ In such cases, differently than what we observe here, one would expect at the onset of the optical excitation a fully thermalized single electronic population of bulk and surface states that subsequently relaxes on a time scale that is solely determined by the strength of electron–phonon scatterings.^[^ ^58^ ^]^

On the contrary, in complex band systems where electron–electron (electron‐phonon) scatterings are not sufficiently strong (weak), the influence of the reduced efficiency of electron–electron scatterings in establishing thermal equilibrium every time a phonon is emitted needs to be taken into account to properly understand the resulting interplay between the thermalization and relaxation pathways.^[^ ^58^ ^]^ Consistent with our present observations, in this situation, as many more electron–electron scatterings are required to establish thermal equilibrium, the overall relaxation process as well as the time scale of energy transfer to the lattice depend on both the electron–phonon and electron–electron scattering strengths.^[^ ^58^ ^]^ While electron–electron scatterings only redistribute energy within the electronic subsystem because the total energy is conserved in the scattering process, the reason for this interdependence is the increased number of electron–electron scatterings that can contribute to the relaxation through energy redistribution between higher‐ and lower‐energy states, resulting in more secondary excitations emitting phonons.

Unlike electron–phonon scatterings, the energy redistribution between SS1, SS2, and BS states via electron–electron scatterings can proceed through interband transitions involving large‐energy transfers, hence providing a larger phase space available for possible transitions. However, the constraints remain that transitions between antiparallel spin configurations are strictly forbidden, and that every electron–electron scattering event occurs under the conservation of total energy and momentum. Due to the large Rashba splitting and the complex alternating spin texture of the bands, these restrictions substantially decrease the amount of possible transitions that are compatible with the stringent selection rules of the second electron involved in the scattering process. Although the effect is less severe than in the case of electron–phonon scatterings due to the larger phase space available, the overall efficiency of interband electron–electron scatterings is significantly reduced and not sufficient to quickly establish a global thermal equilibrium between SS1, SS2, and BS states.

There are several reasons for this behavior. First, there is no efficient thermalization channel in which to redistribute energy between SS1 and SS2 states via low‐*k*‐transfer interband electron–electron scatterings due to the opposite spin texture of the surface Rashba bands (Figure 1f,g). Second, thermalization between SS1 and SS2 states via large‐*k*‐transfer interband electron–electron scatterings is an extremely inefficient process due to the large Rashba splitting and the hole‐like dispersion of the surface bands. It is understood that a large‐*k*‐transfer interband transition reversing the linear electron momentum has a higher probability when the final (initial) energy of the second electron is the same as the initial (final) energy of the first, and that such a process does not contribute to the thermalization between SS1 and SS2 states. Moreover, if the two energies differ, there is essentially no thermalization channel in which this type of interband transitions can redistribute energy between the SS1 and SS2 states under the preservation of the total linear momentum.

The third reason is the reduced efficiency of interband electron–electron scatterings in redistributing energy between surface and bulk states. In part, this is due to the complex alternating spin texture and hole‐like dispersion of the bands (Figure 1f–i), which limit most transitions between SS1 or SS2 and BS states to low‐*k*‐transfer interband electron–electron scatterings. It is important to note that this type of transitions are not completely suppressed by the out‐of‐plane canting of the electron spins (Figure 1g,i), pinpointing the crucial role of spin‐dependent hybridization in the process of interband thermalization between surface and bulk states. However, the restricted phase space accessible for electron scattering into excited bulk states within a narrow energy region close to *E*_F_ further reduces the efficiency of this thermalization channel. This also limits the number of possible interband electron transitions that contribute to global thermalization between surface and bulk states to lower‐energy transfers, as if an excited electron in a higher‐energy SS1 or SS2 state loses a significant amount of energy by scattering with another excited electron within the BS band just above *E*_F_, the two electrons will simply swap bands, but this process will not contribute to the interband thermalization. A direct consequence is a smaller energy redistribution between higher‐ and lower‐energy states through interband transitions where the relaxation of an excited electron via electron–electron scattering is accompanied by the excitation of a second electron into a lower‐energy state, leading to the unusual situation in which energy relaxation starts and even continues throughout interband thermalization (compare, e.g., Figures 3a,b and 2f).

While the large Rashba splitting and the alternating spin texture of the bands are two of the key properties underlying the reduced efficiency of interband electron–electron scatterings, causing a bottleneck in the process of interband thermalization, this is not the case for intraband electron–electron scatterings. The reason is that the main electron–electron scattering events that are responsible for the intraband thermalization involve electron transitions between nearly parallel spin states. It is evident that these type of transitions are more efficient in redistributing energy, and therefore intraband thermalization via electron–electron scatterings is established on a much shorter time scale than interband thermalization, as seen in Figure 3. However, the efficiency with which intraband electron–electron scatterings can redistribute energy, in particular through the relaxation of a higher‐energy excited electron into an empty state slightly above *E*_F_ and the subsequent excitation of another electron from below *E*_F_, again depends on how many states are already filled, or in other words, on the energetic distribution of all other electrons in the vicinity of *E*_F_. In contrast to the spin texture of excited states which is rather independent of lattice temperature, this energetic distribution is likely to be the key for a faster intraband and interband thermalization at lower temperature, because near *E*_F_ is where one can find a lot of filled states. The underlying reason is that when temperature decreases and the energetic distribution becomes very narrow, there are more empty states to scatter into. The effect is much less pronounced in the case of intraband thermalization due to the faster time scales involved and because interband electron–electron scatterings are not efficient in redistributing energy between surface and bulk states.

### 2.5 Contribution of Electron–Electron Scatterings to Energy Relaxation

Having shown that the intertwined thermalization and relaxation pathways of excited bulk and surface Rashba states in α‐GeTe are ultimately determined by electron–electron scattering processes of low efficiency, we now proceed to further understand the influence of these processes on the energy relaxation. Since bulk and surface states behave as continuously thermalizing electronic populations due to the presence of multiple electron–electron scattering events during carrier relaxation, we turn our attention to the temporal evolution of the excited distribution of electrons and holes.

**Figure 4:**
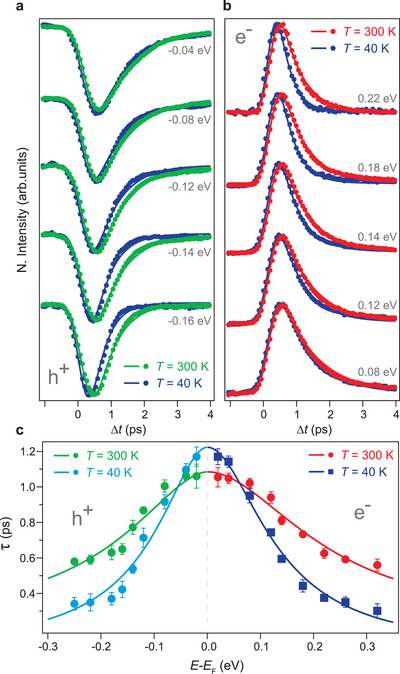
Relaxation dynamics of electrons and holes in α‐GeTe following ultrafast optical excitation. a,b) Temporal evolution of the transient populations of excited carriers at selected energies below (a) and above (b) the Fermi level (*E*_F_). The green (blue) traces in (a) correspond to measurements at a sample temperature of *T* = 300 K (40 K) for excited holes. The red (blue) traces in (b) are the corresponding measurements for excited electrons. The solid lines in (a) and (b) are fits to an exponential decay convolved with a Gaussian profile that accounts for the time resolution. c) Energy dependence of the characteristic decay times for electrons (red circles and blue squares) and holes (green and blue circles) at different temperatures (300 and 40 K). The vertical error bars correspond to ± one standard deviation. The solid lines are a fit to a model which includes the important contribution from electron–electron scatterings to the energy relaxation.

In **Figure** **4**a, we compare the transient photoemission intensities at selected energies below *E*_F_ for excited holes at sample temperatures of *T* = 300 K and 40 K (green and blue traces, respectively). A similar comparison at selected energies above *E*_F_ is displayed in Figure 4b for excited electrons (red and blue traces, respectively). The decrease of intensity and its subsequent recovery in Figure 4a clearly reflects the gain of intensity and its subsequent decay in Figure 4b. The fact that, in both cases, carrier relaxation becomes slower as energy decreases toward *E*_F_ underlines the cascade process of excited electrons and holes that emerges from individual electron–electron and electron–phonon scattering events following optical excitation. However, the fact that the relaxation dynamics becomes faster with decreasing temperature as the energy of excited electrons and holes increases when moving away from *E*_F_, evidences the critical role of electron–electron scatterings in redistributing energy between higher‐ and lower‐energy states. This behavior is consistent with a more important contribution of electron–electron scatterings to the energy relaxation the higher the energy of excited electrons and holes. On the other hand, the smaller differences with decreasing temperature observed at lower energies indicate that electron–phonon scatterings are predominantly responsible for the energy relaxation process in the vicinity of *E*_F_.

To gain more insight into the relative contributions of electron–electron and electron–phonon scatterings to the energy relaxation, in Figure 4c, we compare the energy dependence of the characteristic relaxation times for electrons (red circles and blue squares) and holes (green and blue circles) at different temperatures (300 and 40 K). The shorter carrier relaxation times in the low‐energy region near *E*_F_ at higher temperature are consistent with a time scale of energy transfer to the lattice predominantly associated with electron–phonon scatterings, as also seen in Figure 3a,b. Conversely, the shorter carrier relaxation times at higher energies and lower temperature are consistent with a time scale predominantly associated with energy redistribution between higher‐ and lower‐energy states due to electron–electron scatterings. It is important to note, however, that due to the nature of the cascade process, which causes an excess population of electrons and holes at low energies during interband thermalization, there is an interdependence of both time scales on the relative strengths of the electron–electron and electron–phonon interactions.^[^ ^58^ ^]^

More in detail, we derive that the energy dependence of the relaxation times of electrons and holes can be properly described as $\tau^{-1}=\tau_{0}^{-1}+\alpha |E{|}^{\beta }\ln|E|$ (solid colored lines in Figure 4c). Here, τ_0_ is an effective time constant containing the contributions from electron–phonon scatterings as well as electron transport, and the second term, where there is a logarithmic correction that accounts for the influence of the surface,^[^ ^52^ ^]^ is related to the contribution from electron–electron scatterings.^[^ ^52^, ^56^ ^]^ The parameter α is the characteristic electron–electron scattering constant, which depends on the averaged electron–electron scattering probability, and therefore on the electron–electron interaction strength.^[^ ^56^, ^58^ ^]^

From the symmetric distribution of the relaxation times of electrons and holes at a fixed sample temperature in Figure 4c, we obtain that this model, although somewhat oversimplified, is in good agreement with the experimental data at *T* = 300 K (40 K) with the fitting parameters τ_0_ = 1085 (1224) ± 48 (72) fs, α = 0.011 (0.032) ± 0.004 (0.007) fs^−1^eV^−2^, and β = 1.98 (2.01) ± 0.05 (0.08). The fact that both at room and low temperature τ behaves with β close to the predicted value of 2 highlights the important contribution of electron–electron scatterings to the energy relaxation.^[^ ^52^, ^56^, ^58^ ^]^ This behavior is in stark contrast to that expected in an intraband cooling scenario determined solely by electron–phonon scatterings, a case in which the energy dependence of the relaxation rate should exhibit a perfectly linear behavior as predicted by the two‐temperature model,^[^ ^63^ ^]^ which completely neglects the important role of electron–electron interactions.^[^ ^56^, ^58^ ^]^ Despite its limited applicability, the relaxation rate predicted by the two‐temperature model is usually considered an appropriate descriptor of the electron–phonon interaction strength, γ_ep_.^[^ ^58^ ^]^ Differently from this, in the limit 0.05 < γ_ep_/β_ee_ < 2, the time scale in which electron–electron and electron–phonon interactions influence the rate at which energy is transferred to the lattice can be well approximated as $\tau_{0}^{*}\approx 2.5\times \gamma_{\text{ep}}^{-0.75}\beta_{\text{ee}}^{-0.25}$.^[^ ^58^ ^]^ Here, β_ee_ is the relaxation rate for > 0.3 eV excitations, representing the strength of the electron–electron interactions.^[^ ^58^ ^]^ In our present case, this yields $\beta_{\text{ee}}^{-1}$ ≈ 302 fs at 40 K and $\beta_{\text{ee}}^{-1}$ ≈ 559 fs at 300 K. On the other hand, taking into account an electron–phonon coupling constant λ = 0.55,^[^ ^64^ ^]^ we obtain an elementary electron–phonon scattering time of $\tau_{\text{ep}}^{*}$ ≈ 33 fs at 40 K and $\tau_{\text{ep}}^{*}$ ≈ 22 fs at 300 K. Both values can be approximated by considering that at *T* < *T*_D_, the electron–phonon scattering time is $\tau_{\text{ep}}^{*}$ = 3*ℏ*/2πλ*k*_B_ *T*_D_, where *T*_D_ = 200 K is the Debye temperature.^[^ ^64^ ^]^ This, in conjunction with the small energy of the phonons, yields a two‐temperature model prediction of the relaxation time of $\gamma_{\text{ep}}^{-1}$ ≈ 574 fs at 40 K and $\gamma_{\text{ep}}^{-1}$ ≈ 383 fs at 300 K. Taking altogether, at 40 K, we derive γ_ep_/β_ee_ ≈ 0.5 and $\tau_{0}^{*}$ ≈ 1220 fs, and at 300 K, γ_ep_/β_ee_ ≈ 1.4 and $\tau_{0}^{*}$ ≈ 1053 fs. The fact that, at both temperatures, the values of $\tau_{0}^{*}$ are in excellent quantitative agreement with the fitting parameters of τ_0_ obtained from the energy dependence of the relaxation times in Figure 4c, highlights that our initial model properly captures the complex interplay between electron–electron and electron–phonon scatterings in determining the characteristic time scale of energy transfer to the lattice, with a negligible contribution from electron transport. On the other hand, the quantitative agreement of the characteristic electron–electron scattering constant α, which can be properly described as α ≈ β_ee_/(0.3eV)^2^ at both temperatures, confirms that the relaxation of higher‐energy excitations is insensitive to the strength of the electron–phonon interactions. Therefore, this time scale is instead entirely determined by electron–electron scatterings contrary to what would be expected in a limit where γ_ep_/β_ee_  >> 1.^[^ ^58^ ^]^

### 2.6 Complete Disentanglement of All Possible Scattering Channels of Ultrafast Energy Transfer

Finally, in order to identify which individual scattering channels are predominantly responsible for the contrasting temperature dependence of the thermalization pathways within the electronic subsystem and between the electronic subsystem and the lattice, we performed theoretical calculations within the Boltzmann approach including all types of elementary scatterings.^[^ ^66^, ^67^, ^68^ ^]^ Among several existing state‐of‐the‐art theoretical methods that have proven successful for describing nonequilibrium dynamics of excited states, such as real‐time time‐dependent density‐functional theory (RTTD‐DFT)^[^ ^69^ ^]^ and other types of nonequilibrium DFT calculations,^[^ ^70^ ^]^ the Boltzmann approach is a relatively inexpensive and very powerful method to identify in detail which individual scattering channels drive the different thermalization pathways.^[^ ^66^, ^67^, ^68^ ^]^ To this end, we computed the temperature dependence of the scattering rates for all electron–electron and electron–phonon scatterings that can be obtained from the Boltzmann scattering equation when combining the bulk (BS) and surface (SS1, SS2) transient Rashba bands with a single effective phonon band. The calculations of the scattering rates account for all potential spin‐dependent intraband and interband transitions under any given scattering angle in energy–momentum space.

The results for the expressions of the scattering rates associated with any possible individual scattering channel are given in the Supporting Information. They are obtained as functional derivatives of the corresponding scattering integral with respect to one of the populations calculated at thermal equilibrium (see Supporting Information Note S1). The resulting electron–electron (electron–phonon) scattering integrals are in eight (six) dimensions and include three Dirac delta functions representing momentum and energy conservation. The integrals are performed numerically using a recently developed technique to solve the full time‐dependent Boltzmann scattering integral without any close‐to‐equilibrium approximation and with simultaneous momentum, energy, and particle conservation^[^ ^66^, ^67^, ^68^ ^]^ (see Experimental Section and Supporting Information Note S2 for more details).

In order to substantially reduce the complexity of the problem, we assume cylindrical symmetry and use the experimental band dispersions as reference for the input of the calculations (see Experimental Section). To this end, we assign a chiral spin texture with a helicity corresponding to that in Figure 1 to each band. For further simplicity, we assume a single optical Einstein‐like phonon band (Ph) with an energy of *ϵ*_Ph_ = 0.01 eV. Moreover, while the spin overlap entering the scattering matrix elements is included as dependent on all the involved momenta, the spatial component of the matrix elements is assumed constant across all the scattering channels.

Let us now focus on the interband thermalization time scale and compute the temperature dependence of the scattering rates of all possible electron–electron scatterings. For instance, there are 14 relevant independent scattering channels involving the outer surface Rashba band (SS1) which allow for interband energy transfer. These are listed in the central column of **Table** **1** and are numbered in accordance with their maximum scattering rate from high to low. The right hand column in Table 1 indicates how the scattering rates are affected by the increasing of lattice temperature when integrating over the entire band dispersion, with upward (downward) facing arrows demonstrating a higher (lower) scattering rate at 300 K than at 40 K. We observe that the strongest scattering channels allowing for interband energy transfer become weaker with increasing temperature, therefore behaving consistently with the slower interband thermalization observed experimentally, as discussed in the previous sections. We can conclude, therefore, that these few dominant scattering channels permitting interband energy transfer primarily dictate the overall interband thermalization process in the experimental picture.

**Table 1:** Relevant electron–electron scattering channels of ultrafast energy transfer in α‐GeTe. The most important electron–electron scattering channels responsible for interband energy transfer between bulk (BS) and surface (SS1, SS2) states are identified. The individual scattering channels, which are predominantly associated with interband transitions involving SS1 states, are ordered by decreasing extrema of the scattering rate within the energy–momentum range occupied by the relevant bands. For example, channel 4 has the fourth shortest scattering time, and describes an excited electron originating in SS1 that relaxes to a lower‐energy state within SS1. Consequently, a secondary electron is excited from SS2 to a higher energy within BS. The final column indicates whether the gradient Δ*R*/Δ*T* (*R*, is the scattering rate) in the energy region dominated by electron–electron scatterings is negative or positive, or, in other words, whether the scattering rate at a temperature *T* = 300 K is higher (↗) or lower (↘) than at 40 K.

ID	e^−^ −e^−^ scattering	Δ*R*/Δ*T*
1	SS1 + SS1 ↔ SS1 + BS	↘
2	SS1 + SS2 ↔ SS1 + SS2	↘
3	SS1 + BS ↔ SS2 + SS2	↘
4	SS1 + SS2 ↔ SS1 + BS	↘
5	SS1 + SS2 ↔ SS2 + SS2	↘
6	SS1 + BS ↔ BS + BS	↗
7	SS1 + BS ↔ SS2 + BS	↗
8	SS1 + BS ↔ SS1 + BS	↗
9	SS1 + SS2 ↔ BS + BS	↗
10	SS1 + SS2 ↔ SS2 + BS	↗
11	SS1 + SS1 ↔ BS + BS	↗
12	SS1 + SS1 ↔ SS2 + BS	↗
13	SS1 + SS1 ↔ SS2 + SS2	↗
14	SS1 + SS1 ↔ SS1 + SS2	↗

**Figure 5:**
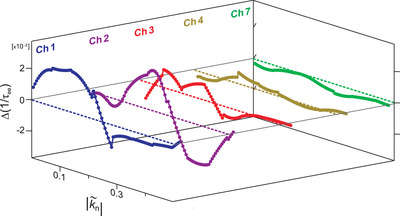
Calculated temperature dependence of the dominant electron–electron scattering channels as a function of band position. Full band‐position dependence of select electron–electron scattering channels involving SS1 states, as described in Table 1. The variable $\left|{\tilde{k}}_{n}\right|$ indicates the considered energy–momentum point along the band in units of Å^−1^, with the higher‐energy region dominated by electron–electron scatterings at $\left|{\tilde{k}}_{n}\right|$ < 0.3 Å^−1^. The traces are obtained from the difference in the calculated scattering rates as determined at 40 and 300 K. A positive value represents faster interband thermalization with decreasing temperature. The legend indicates the corresponding scattering channel in Table 1.

We further demonstrate this by computing these temperature‐dependent scattering rates along the band dispersion (denoted as ${\tilde{k}}_{n}(E)$). In **Figure** **5**, we display the absolute difference between these scattering rates at 40 and 300 K for selected scattering channels in Table 1. From this, and by careful comparison to the transient electronic structure in Figure 3c, it can be concluded that the overall reduction of the scattering rates for the dominant channels derive from the reduction of the transition joint density of states, due to the increased thermal population of states above *E*_F_. The underlying reason for this behavior is the large Rashba splitting of the bands. We note that the additional band position dependence of these lattice temperature dependent scattering rates observed here is driven predominantly by the changing of energy as the band disperses toward higher ${\tilde{k}}_{n}$, and not by the changing of *k*_∥_ itself, in‐line with the previous experimental discussions. Next, we turn to disentangling the scattering channels primarily associated with energy transfer from the electronic degrees of freedom to the phononic system. In analogy to the above discussion, in order to derive the dominant mechanisms for the experimentally observed temperature dependence of this energy‐transfer process, we construct all possible electron–phonon scatterings, listed in Table S1, Supporting Information. In this case, all relevant scattering channels show a more conventional behavior, increasing in strength with increasing lattice temperature, again in direct correspondence to the experimental picture.

## 3 Conclusion

We have used femtosecond time‐ and angle‐resolved photoemission spectroscopy to directly access the relevant mechanisms that enable ultrafast energy transfer through nonequilibrium transient Rashba states in the ferroelectric Rashba semiconductor α‐GeTe. Our experimental findings reveal a strong impact of Rashba‐type spin–orbit effects on the thermalization processes that govern the time scales of ultrafast energy transfer following optical excitation. We have demonstrated how the Rashba effect gives rise to a complex interdependence of these times scales on the relative strength of both electron–electron and electron–phonon interactions, with spin‐selection constrained electron–electron scatterings found both to dominate dynamics away from the immediate vicinity of the Fermi level and to weaken with increasing temperature. We have shown how this behavior is responsible for the observation of an unusual temperature dependence of the thermalization pathways that is ultimately related to the reduced efficiency of interband electron–electron scatterings caused by the Rashba effect, both at the surface and in the bulk. By solving the full, non‐linearized, time‐dependent Boltzmann equation, we have confirmed the microscopic mechanism of the experimental findings and explicitly shown the opposite behavior of all relevant electron–electron and electron–phonon scattering channels with temperature. The present findings taken altogether provide clear evidence for the decisive role of the Rashba effect in determining the fundamental processes that control the time scales of ultrafast information processing based on the electron spin, which is highly relevant for potential applications of ferroelectric Rashba semiconductors and their excitations in ultrafast spintronics.

## 4 Experimental Section

### Photoemission Experiments

The photoemission experiments were carried out at the spin‐resolved ARPES station permanently installed at the U125‐2‐PGM beamline of BESSY‐II in Helmholtz‐Zentrum Berlin. The tr‐ARPES measurements were performed stroboscopically using pump (1.5 eV) and probe (6 eV) femtosecond (fs) pulses from a Ti: sapphire oscillator coupled to an ultrafast amplifier laser system (RegA, Coherent). The pulses impinged the sample under an angle of 45°, and the pump–probe time delay Δ*t* was varied using a optical delay stage. Measurements at 11 eV were carried out using synchrotron light incident on the sample under the same geometry. The repetition rate of the laser was 150 kHz. The time resolution was ≈160 fs, and the pump fluence ≈100 μJcm^−2^. The base pressure of the photoemission setup was better than 1 × 10^−10^ mbar. The 0.5 μm‐thick α‐GeTe(111) films were grown by molecular beam epitaxy on BaF_2_(111) substrates in a different chamber, and transported to the photoemission setup inside an ultrahigh vacuum suitcase (Ferrovac GmbH) at pressures below 5 × 10^−10^ mbar to avoid contamination. Emitted photoelectrons were detected with a Scienta R4000 electron analyzer, and the angular and energy resolutions were set to 0.1° and 5 meV, respectively. For spin analysis, a Rice University Mott‐type spin polarimeter was used, operated at 25 kV and capable of detecting both in‐plane and out‐of‐plane components of the spin polarization. Resolutions of spin‐resolved ARPES measurements were 0.75° (angular) and 80 meV (energy).

### Theoretical Calculations

Theoretical calculations were performed within the Boltzmann approach taking into account all types of elementary scatterings. To calculate the temperature dependence of the scattering rates associated with any possible individual scattering channel, the Boltzmann scattering equation was used. The calculations were carried out using a recently developed numerical technique to solve the full, non‐linearized, time‐dependent Boltzmann scattering equation with no close to equilibrium approximations and simultaneous particle, momentum, and energy conservation.^[^ ^66^, ^67^, ^68^ ^]^ The method uses piecewise continuous polynomial basis functions for populations, dispersions, and scattering matrix elements, and a hybrid analytical inversion and Monte Carlo integration for the scattering integrals. For the input of the calculations, the dispersions of the three electronic bands SS1, SS2, and BS were discretized by assuming cylindrical symmetry and the experimental band dispersions in Figure 1 and ref. [48]. The scattering rates for each band and for each scattering channel were obtained by taking the functional derivative with respect to the corresponding population,^[^ ^66^ ^]^ and integrated numerically using the above mentioned technique. Spin selection rules for all possible electron–electron and electron–phonon scatterings were explicitly included in the functional form of the constrained scattering amplitude associated with each individual scattering channel. The scattering matrix elements were considered dependent on all the momenta of the states involved in the transition (e.g., in the case of any possible four‐leg electron–electron scattering channel), and constructed by including the momenta‐dependent spin overlap and a simplified treatment of the spatial component based on a Yukawa potential between free‐particle‐like Bloch states (see Supporting Information).

## Conflict of Interest

The authors declare no conflict of interest.

## Author Contributions

O.J.C., I.W., and F.F. contributed equally to this work. O.J.C., F.F. and J.S.‐B. performed photoemission experiments. I.W. and M.B. performed theoretical calculations. G.S. performed sample preparation and preliminary characterization. O.J.C. and J.S.‐B. performed data analysis and draft planning. J.S.‐B. wrote the manuscript with input from all co‐authors. M.B. was responsible for the coordination of the theoretical work. J.S.‐B. was responsible for the conception and the overall direction of this work.

## Supporting information

Supporting Information

## Data Availability

The data that support the findings of this study are available from the corresponding author upon reasonable request.
