# Supplementary material for: Ultrafast Thermalization Pathways of Excited Bulk and Surface States in the Ferroelectric Rashba Semiconductor GeTe
Source: Adv Mater. 2022 May 12;34(24):2200323. doi: 10.1002/adma.202200323 (PMC11475237; doi:10.1002/adma.202200323)
Supplement: Supplementary file 1 — Supporting Information [file ADMA-34-2200323-s001.pdf]

ADVANCED MATERIALS

Supporting Information

for *Adv. Mater.,* DOI: 10.1002/adma.202200323

Ultrafast Thermalization Pathways of Excited Bulk and Surface States in the Ferroelectric Rashba Semiconductor GeTe

Oliver J. Clark, Indrajit Wadgaonkar, Friedrich Freyse, Gunther Springholz, Marco Battiato, and Jaime Sánchez-Barriga**

Supplementary Information

Ultrafast thermalization pathways of excited bulk and surface states in the ferroelectric Rashba semiconductor GeTe

O. J. Clark,[1,†] I. Wadgaonkar,[2,†] F. Freyse,[1,3,†] G. Springholz,[4] M. Battiato,[2,*] and J. Sánchez-Barriga[1,5,*]

[1]*Helmholtz-Zentrum Berlin für Materialien und Energie, Elektronenspeicherring BESSY II, Albert-Einstein-Str. 15, 12489 Berlin, Germany*

[2]*Nanyang Technological University, Nanyang Link 21, 637371, Singapore*

[3]*Institut für Physik und Astronomie, Universität Potsdam, Karl-Liebknecht-Str. 24/25, 14476 Potsdam, Germany*

[4]*Institut für Halbleiter- und Festkörperphysik, Johannes Kepler Universität, A-4040 Linz, Austria*

[5]*IMDEA Nanoscience, C/ Faraday 9, Campus de Cantoblanco, 28049, Madrid, Spain*

Supplementary Note 1: Scattering rates integrals

The scattering operator of the time dependent Boltzmann Equation [1, 2] is the sum of a number of scattering integrals pertaining to different scattering channels. The scattering integrals depend on the momentum-resolved populations of all the states involved in the transition. The functional derivative of a given scattering integral with respect to one of the populations provides the momentum-resolved scattering rate, i.e. the inverse lifetime, of that state with respect to that transition [3]. For instance, the scattering rate for an electron-electron scattering (the case of electron-phonon scattering can be easily constructed following the same structure) is written as

$$\frac{1}{\tau_{n_0}(\mathbf{k}_0)} = \sum_{\mathbf{G}} \iiint_{V_{BZ}^3} d\mathbf{k}_1 \, d\mathbf{k}_2 \, d\mathbf{k}_3 \;\; w_{0123}^{e-e} \;\; \delta(\epsilon_0 + \epsilon_1 - \epsilon_2 - \epsilon_3) \\ \delta(\mathbf{k}_0 + \mathbf{k}_1 - \mathbf{k}_2 - \mathbf{k}_3 + \mathbf{G})[(1 - f_1^{eq})f_2^{eq}f_3^{eq} - f_1^{eq}(1 - f_2^{eq})(1 - f_3^{eq})] \tag{1}$$

where $\epsilon_i = \epsilon_{n_i}(\mathbf{k}_i)$ is the dispersion in the band n_i, $f_i^{eq} = f_{FD}(\epsilon_{n_i}(\mathbf{k}_i))$ is the equilibrium population (a Fermi-Dirac f_{FD} for electrons and a Bose-Einstein in case of phonons), and $\mathbf{G}$ are the possible umklapp vectors.

The term $w_{0123}^{e-e} = x_{n_0+n_1 \leftrightarrow n_2+n_3}^{e-e}(\mathbf{k}_0, \mathbf{k}_1, \mathbf{k}_2, \mathbf{k}_3)$ is the scattering matrix element. {0,1,2,3} are labels for the participating electron states in the considered four-leg scattering channel. Assuming that the corresponding wave functions are the product of a spatial and a spin part, and that the scattering operator is spin diagonal, the scattering matrix elements are a product of a spatial and a spin part. For simplicity, we do not calculate the spatial part of w_{0123}^{e-e} and assume it constant across all scattering channels. The spin overlap instead can be calculated analytically using the momentum-dependent spin directions associated with the Rashba spin texture of the bands. We postpone the derivation of the specific functional form of the scattering amplitude used in our analysis to Supplementary Note 2 .

For the representative channel 0 described above, the scattering amplitude takes the following functional form,

$$w_{0123}^{e-e}(\mathbf{k}_0, \mathbf{k}_1, \mathbf{k}_2, \mathbf{k}_3) = W \Bigg[\cos\left(\frac{(1 - \eta_1 * \eta_3) * \pi}{4} - \frac{\theta_1 - \theta_3}{2}\right) \cos\left(\frac{(1 - \eta_0 * \eta_2) * \pi}{4} - \frac{\theta_0 - \theta_2}{2}\right) \\ - \cos\left(\frac{(1 - \eta_0 * \eta_3) * \pi}{4} - \frac{\theta_0 - \theta_3}{2}\right) \cos\left(\frac{(1 - \eta_1 * \eta_2) * \pi}{4} - \frac{\theta_1 - \theta_2}{2}\right) \Bigg]^2 \tag{2}$$

where, W is an adjustable parameter in our model and $\vec{k}_{i,j,k,l}$ are the momentum labels of the participating electron states. $\theta_{i,j,k,l}$ are the corresponding chiral spin orientations defined as $\theta = \tan^{-1}(k_y/k_x)$.

Supplementary Note 2: Derivation of the functional form of the scattering amplitude

We report the derivation of the analytic expression for the scattering matrix element in the case of a scattering involving four electronic states. The expression for the case of electron-phonon scattering can be constructed similarly.

We assume the single-electron wave function as a product of a spatial part $\langle\phi(\mathbf{r})|$ and a spin part $\langle s(\sigma)|$. In our case the spatial part depends on the momentum $\mathbf{k}$ and on the band index n. We however suppress the explicit dependence on n for shortness $\langle\phi_{\mathbf{k}}(\mathbf{r})|$. The spin part is also momentum dependent, with a chiral spin component associated with an helicity $\eta = \pm 1$ in each band: $\langle s_{\mathbf{k},\eta}(\sigma)|$. We also assume that the two-electron wave function is simply the antisymmetrized product of the two single-particle wave functions

$$\frac{1}{\sqrt{2}}\left(\langle\phi_{\mathbf{k}_1}(\mathbf{r}_A)|\,\langle s_{\mathbf{k}_1,\eta_1}(\sigma_A)|\,\langle\phi_{\mathbf{k}_2}(\mathbf{r}_B)|\,\langle s_{\mathbf{k}_2,\eta_2}(\sigma_B)| - \langle\phi_{\mathbf{k}_2}(\mathbf{r}_A)|\,\langle s_{\mathbf{k}_2,\eta_2}(\sigma_A)|\,\langle\phi_{\mathbf{k}_1}(\mathbf{r}_B)|\,\langle s_{\mathbf{k}_1,\eta_1}(\sigma_B)|\right) \tag{3}$$

where A and B refer to different particles and the integer numbers to the different states.

Assuming the interaction hamiltonian ΔH_{int} as spin independent, the scattering matrix element reduces to

$$\begin{aligned} &w^{e-e}_{0123}(\mathbf{k}_0,\mathbf{k}_1,\mathbf{k}_2,\mathbf{k}_3) = \\ &\Big|\,\langle\phi_{\mathbf{k}_2}(\mathbf{r}_A)\phi_{\mathbf{k}_3}(\mathbf{r}_B)\,|\Delta H_{int}|\,\phi_{\mathbf{k}_0}(\mathbf{r}_A)\phi_{\mathbf{k}_1}(\mathbf{r}_B)\rangle\,\langle s_{\mathbf{k}_3,\eta_3}|s_{\mathbf{k}_1,\eta_1}\rangle\,\langle s_{\mathbf{k}_2,\eta_2}|s_{\mathbf{k}_0,\eta_0}\rangle - \\ &\langle\phi_{\mathbf{k}_2}(\mathbf{r}_A)\phi_{\mathbf{k}_3}(\mathbf{r}_B)\,|\Delta H_{int}|\,\phi_{\mathbf{k}_1}(\mathbf{r}_A)\phi_{\mathbf{k}_0}(\mathbf{r}_B)\rangle\,\langle s_{\mathbf{k}_3,\eta_3}|s_{\mathbf{k}_0,\eta_0}\rangle\,\langle s_{\mathbf{k}_2,\eta_2}|s_{\mathbf{k}_1,\eta_1}\rangle\,\Big|^2 \end{aligned} \tag{4}$$

where spin coordinates have been dropped and the bracket notation contracted, for shortness.

- Spatial Matrix elements:

In the following we derive the expression for the spatial part of the matrix element in Eq. 4. Considering $|\phi_{\mathbf{k}_n}\rangle$ as a Bloch eigenstate we have $\langle\mathbf{r}|\phi_{\mathbf{k}_n}\rangle = e^{i\mathbf{k}_n\mathbf{r}}\,u_{\mathbf{k}_n}(\mathbf{r})$ where the function $u_{\mathbf{k}_n}(\mathbf{r})$ has the lattice periodicity. The spatial matrix element becomes

$$\begin{aligned} &\langle\phi_{\mathbf{k}_2}(\mathbf{r}_A)\phi_{\mathbf{k}_3}(\mathbf{r}_B)|\,\Delta H_{int}\,|\phi_{k_0}(\mathbf{r}_A)\phi_{k_1}(\mathbf{r}_B)\rangle \\ &= \iint \Delta H_{int}(\mathbf{r}_A,\mathbf{r}_B)e^{-i(\mathbf{k}_2-\mathbf{k}_0)\mathbf{r}_A}e^{-i(\mathbf{k}_3-\mathbf{k}_1)\mathbf{r}_B}\; u^*_{\mathbf{k}_2}(\mathbf{r}_A)u_{\mathbf{k}_0}(\mathbf{r}_A)u^*_{\mathbf{k}_3}(\mathbf{r}_B)u_{\mathbf{k}_1}(\mathbf{r}_B)\; d\mathbf{r}_A d\mathbf{r}_B \end{aligned}$$

where we remind that $u_{\mathbf{k}_n}$ is dependent on the suppressed band index as well. We now assume that the product of the u functions can be approximated by a band and momenta independent constant U, the remaining integral is simply the two-dimensional Fourier transform of the function ΔH_{int}. We make the further simplifying assumption that the interaction hamiltonian is the Yukawa potential with screening length λ and charge e and obtain

$$\langle \phi_{\mathbf{k}_2}(\mathbf{r}_A)\phi_{\mathbf{k}_3}(\mathbf{r}_B)|\,\Delta H_{int}\,|\phi_{\mathbf{k}_0}(\mathbf{r}_A)\phi_{\mathbf{k}_1}(\mathbf{r}_B)\rangle = \frac{16\pi^2 Ze^2}{|\mathbf{k}_2-\mathbf{k}_0|^2+\lambda^2}\delta\left(\mathbf{k}_0+\mathbf{k}_1-\mathbf{k}_2-\mathbf{k}_3\right)$$

- Spin overlap:

We have assumed that the spin orientation at a given momentum $\mathbf{k}$ is defined by a chiral spin texture that depends on the helicity η in each band. We have

$$|s_{\mathbf{k},\eta=+1}\rangle = \begin{pmatrix}\cos(\theta/2)\\ \sin(\theta/2)\end{pmatrix} \qquad \text{and} \qquad |s_{\mathbf{k},\eta=-1}\rangle = \begin{pmatrix}\sin(\theta/2)\\ -\cos(\theta/2)\end{pmatrix}$$

where $\tan\theta = k_y/k_x$ and $\mathbf{k} = \{k_x, k_y\}$.

The spin overlap is obtained as

$$\left\langle s_{\mathbf{k}_i,\eta_i}|s_{\mathbf{k}_j,\eta_j}\right\rangle = \cos\left(\frac{(1-\eta_i*\eta_j)*\pi}{4} - \frac{\theta_j-\theta_i}{2}\right)$$

- Functional form of the scattering amplitude:

Taking altogether, the full matrix element in Eq.4 becomes

$$\begin{aligned} w^{e-e}_{0123}(\mathbf{k}_0,\mathbf{k}_1,\mathbf{k}_2,\mathbf{k}_3) &= \delta\left(\mathbf{k}_0+\mathbf{k}_1-\mathbf{k}_2-\mathbf{k}_3\right)\\ \Bigg[&\frac{16\pi^2 Ze^2}{|\mathbf{k}_2-\mathbf{k}_0|^2+\lambda^2}\cos\left(\frac{(1-\eta_1*\eta_3)*\pi}{4}-\frac{\theta_1-\theta_3}{2}\right)\cos\left(\frac{(1-\eta_0*\eta_2)*\pi}{4}-\frac{\theta_0-\theta_2}{2}\right)\\ &-\frac{16\pi^2 Ze^2}{|\mathbf{k}_2-\mathbf{k}_1|^2+\lambda^2}\cos\left(\frac{(1-\eta_0*\eta_3)*\pi}{4}-\frac{\theta_0-\theta_3}{2}\right)\cos\left(\frac{(1-\eta_1*\eta_2)*\pi}{4}-\frac{\theta_1-\theta_2}{2}\right)\Bigg]^2 \end{aligned}$$

The parameter Z is an absolute rescaling of all scattering rates that does not affect the temperature dependence. We have tested the influence of the screening length λ and found no differences in the overall qualitative behavior with temperature. Finally, we have verified that our results are independent of different parametrizations of the Yukawa potential. This ensures that our calculations are robust and independent of the spatial approximation used.

ID	e^{-}– Ph scattering	$\Delta R/\Delta T$
1	BS + Ph $\leftrightarrow$ BS	$\nearrow$
2	BS + Ph $\leftrightarrow$ SS2	$\nearrow$
3	BS + Ph $\leftrightarrow$ SS1	$\nearrow$
4	SS2 + Ph $\leftrightarrow$ BS	$\nearrow$
5	SS2 + Ph $\leftrightarrow$ SS2	$\nearrow$
6	SS2 + Ph $\leftrightarrow$ SS1	$\nearrow$
7	SS1 + Ph $\leftrightarrow$ BS	$\nearrow$
8	SS1 + Ph $\leftrightarrow$ SS2	$\nearrow$
9	SS1 + Ph $\leftrightarrow$ SS1	$\nearrow$

TABLE S1. **Relevant electron-phonon scattering channels of energy transfer to the lattice in α-GeTe.** All the important electron-phonon scattering channels responsible for energy transfer from the electronic degrees of freedom to the phononic system show a more conventional behavior with increasing temperature. These electron-phonon scattering channels dominate the overall relaxation process of excited bulk (BS) and surface states (SS1 and SS2) in the vicinity of the Fermi level at low energies. Each individual scattering channel is associated with a different transition, and increases in strength with increasing temperature. The latter is indicated in the final column by the calculated negative gradient $\Delta R/\Delta T$ of the scattering rates as temperature increases, which corresponds to a higher ($\nearrow$) electron-phonon scattering rate at a temperature T= 300 K than at 40 K for each individual scattering channel.

† These authors contributed equally to this work.

* Corresponding authors. E-mails: marco.battiato@ntu.edu.sg; jaime.sanchez-barriga@helmholtz-berlin.de

[1] D. W. Snoke, *Solid state physics: Essential concepts* (Cambridge University Press, 2020).

[2] D. W. Snoke, Ann. Phys. **523**, 87 (2011).

[3] M. Wais, K. Held, and M. Battiato, Comput. Phys. Commun. **264**, 107877 (2021).
